# lncRNA PAPPA-AS1 Induces the Development of Hypertrophic Scar by Upregulating TLR4 through Interacting with TAF15

**DOI:** 10.1155/2021/3170261

**Published:** 2021-07-03

**Authors:** Pengju Fan, Yongjie Wang, Jingjing Li, Man Fang

**Affiliations:** Department of Burn and Plastic, Xiangya Hospital, Central South University, Changsha, Hunan 410008, China

## Abstract

Hypertrophic scar (HTS) is a complicated pathological process induced mainly by burns and wounds, with abnormal proliferation of fibroblasts and the transformation of fibroblasts to myofibroblasts. PAPPA-AS1, a differentially expressed long noncoding RNA (lncRNA) in the HTS tissues, attracted our interests in its potential role and mechanism in the development and process of HTS. In the present study, the regulatory effect of lncRNA PAPPA-AS1 on the Toll-like receptor 4 (TLR4) signal pathway, as well as the molecular mechanism, was investigated. Bioinformatics analysis was utilized to screen the differentially expressed lncRNAs in HTS tissues. PAPPA-AS1 was significantly upregulated in both HTS tissues and hypertrophic scar fibroblast (HTsFb) cells. The expression levels of TLR4, MyD88, TGF-*β*1, collagen I, collagen III, and *α*-SMA were greatly elevated in HTsFb cells. By knocking down PAPPA-AS1, the proliferation of HTsFb cells, TLR4, and TGF-*β*1 signal pathway and the expression of fibrosis markers both in HTsFb cells and HTS tissues were suppressed. It was accompanied by the alleviated pathological state in the HTS tissues, which were significantly reversed by cotransfecting with the pcDNA3.1-TLR4 vector. Positive correlation and interaction were observed between PAPPA-AS1 and TAF15 and between TAF15 and the promoter of TLR4, respectively. In conclusion, lncRNA PAPPA-AS1 might induce the development of HTS by upregulating TLR4 through interacting with TAF15.

## 1. Introduction

Hypertrophic scar (HTS) is the complication of burns or wounds, which is mainly clinically characterized with local redness, itchy pain, hyperplasia becoming hard, local contracture, articular dyskinesia, and even repeated rupturing or cancerization [1, 2]. Not only the normal physiological function and appearance but also the psychology of the patients are significantly influenced by HTS, which contributes to the impaired social interaction and is generally considered as a public health issue. The pathological characteristics of HTS are the abnormal proliferation of fibroblasts, the transformation of fibroblasts to myofibroblasts, and the excessive accumulation of the extracellular matrix (ECM) [3, 4]. Although multiple hypotheses have been proposed to claim the pathogenesis of HTS, the pathological mechanism of HTS still remains unknown. It is of great significance to explore the possible molecular mechanism underlying HTS for the clinical treatment of HTS.

Epigenetics is a biological research method investigating the changes in phenotype that are not rooted in DNA sequence, including the DNA methylation, histone modification, chromatin remodeling, and noncoding RNA [5]. lncRNAs are noncoding RNAs greater than 200 nucleotides in length, which are originally regarded as the “noise” in the genome transcription without biological functions. However, in recent years, lncRNAs are reported to be involved in multiple cellular biological processes, such as epigenetic, transcriptional, and posttranscriptional regulation of gene expression, the silence of X chromosome, the activation of transcription, and nuclear transmission [6–8]. Accumulative evidences suggested the significant function of lncRNA in the process and development of HTS and fibrosis [9–11]. In our preliminary experiment, we found that lncRNA PAPPA-AS1 was differentially expressed in the HTS tissues and normal skin tissues based on the bioinformatics analysis. lncRNA PAPPA-AS1 is a rarely studied lncRNA which was only discussed in adenovirus infection [12], placenta percreta [13], and osteoporosis [14], with very limited information in its properties. It attracted our interest in its potential role and mechanism in the development and process of HTS.

Toll-like receptors (TLRs) are a couple of transmembrane proteins located on the cytomembrane, which recognize the conservative pathogen-associated molecular patterns (PAMPs). It includes lipopolysaccharide (LPS), bacterial lipoprotein, CpG DNA, virus double-strain RNA, hyaluronic acid, heat shock protein (HSPs), and S100 protein [15]. Multiple internal and external substances, such as taxol, fibulin, hyaluronic acid, oligosaccharide, HSP60, HSP70, fibrinogen, and High-mobility group box 1 (HMGB1), can be recognized by TLR4, making TLR4 a promising target in the field of antivirus, antibacteria, and tissue repairment [16–18]. Seki reported that the fake receptor of transforming growth factor-beta (TGF-*β*), bone morphogenetic protein, and activin membrane-bound inhibitor (BAMBI) could be downregulated by stimulating the TLR4 on the membrane of hepatic stellate cells, which further activates the TGF-*β*1 signal pathway and contributes to the liver fibrosis [19].

In the injured skin tissues and lung tissues of scleroderma patients, the abnormal expression of TLR4 activated the Smad signal pathway in the fibroblasts, enhanced the sensitivity of fibroblasts to TGF-*β*1, accelerated the synthesis of collagens, and regulated the expression of several genes involved in the tissue remodeling and extracellular matrix homeostasis [20]. Recently, it was reported that the expression level of TLR4 was relatively higher in the HTS tissues than in the normal skin tissues [21]. The development and process of HTS could be accelerated by activating TLR4 through upregulating TGF-*β*1, connective tissue growth factor, collagen I, and collagen III [22–24], which was consistent with the data achieved in our previous researches [25, 26]. However, the upstream mechanism of TLR4 in the pathogenesis of HTS still remains unknown.

In our preliminary experiments, we found that PAPPA-AS1 could bind with TLR4 in the HTS tissues, which further encouraged us to explore the roles and effect of PAPPA-AS1 and its possible target of TLR4 in the development of HTS. In the present study, the differentially expressed lncRNA in the HTS tissues and normal skin tissues will be screened and the potential biological function of PAPPA-AS1 in the development and process of HTS will be explored by investigating the correlation between PAPPA-AS1 and TLR4.

## 2. Materials and Methods

### 2.1. Bioinformatics Analysis on the HTS Tissues and Normal Skin Tissues

The data of lncRNA and mRNA expression was obtained from the Gene Expression Omnibus (GEO) database repository, the publicly available genomics database from the National Center for Biotechnology Information (NCBI). Dataset GSE151153 has 3 HTS samples and 3 normal samples. Dataset GSE158155 has 24 HTS samples and 4 normal samples. Dataset GSE151153 and dataset GSE158155 were standardized by the RMA algorithm using the limma package in the *R* computing environment, respectively. Next, differential analysis (∣logFC | >2, adjusted *p* value < 0.05) were identified severally. The volcano map was displayed. The correlation analysis on coexpression patterns of differentially expressed lncRNA and mRNA was performed by the pheatmap package, and a heat map was drawn. Gene Ontology (Go) and Kyoto Encyclopedia of Genes and Genomes (KEGG) enrichment analyses were performed on the differentially expressed mRNA of GSE158155 using the function of clusterProfiler in *R*, and the significant pathways were shown, respectively (adjusted *p* value < 0.05).

### 2.2. Animals, Cells, and Treatments

48 BALB/c nude mice (6 weeks old, 20–23 g each) were randomly divided into 6 groups, half male and half female in each group. Mice were purchased from Beijing Vital River Laboratory Animal Technology Co. Ltd. The environmental temperature was maintained at 24–26°C, and the relative humidity was maintained at 50%–60%. All mice were freely ingested water and food. Normal human skin fibroblasts (NsFb) were obtained from Wuhan University Cell Bank, and human HTsFb cells were purchased from Shanghai Bailey Biotechnology Co. Ltd. They were incubated in the completed DMEM medium containing 10% fetal bovine serum (FBS) at 37°C with 5% CO_2_.

### 2.3. Transfection

The HTsFb cells were collected and incubated in 6-well plates. Short hairpin RNAs (shRNAs) against lncRNA PAPPA-AS1 (sh-PAPPA-AS1#1 and sh-PAPPA-AS1#2) were utilized to reduce the PAPPA-AS1. The corresponding negative control was sh-NC (sh-NC). The expression level of lncRNA PAPPA-AS1 was promoted by transfecting with the pcDNA3.1-PAPPA-AS1 vector. PcDNA3.1-NC was used as negative control. And TLR4 was upregulated by transfecting with the pcDNA3.1-TLR4 vector (2.5 *μ*g). The negative control of the two overexpression plasmids was pcDNA3.1-NC. TAF15 was downregulated by transfecting with sh-TAF15 and upregulated by transfecting with over-TAF15. Their negative controls were sh-NC and over-NC, respectively. All the vectors (2.5 *μ*g) were transfected into the HTsFb cells accompanied with lipofectamine 3000 (5 *μ*L, Invitrogen, CA, USA), followed by being harvested at 37°C with 5% CO_2_ after 48 hours of incubation. All the plasmids were synthesized by HonorGene (Changsha, China).

### 2.4. Lentiviral Transfection

Sh-NC, sh-PAPPA-AS1#1, sh-PAPPA-AS1#2, pcDNA3.1-NC, and pcDNA3.1-TLR4 were inserted into lentiviral vector pLVX-IRES-tdTomato. The constructed plasmid and packaging plasmid were cotransfected into HTsFb cells. The virus particles were harvested by ultracentrifugation 48 hours after transfection. Finally, the prepared virus particles were injected into the mice.

### 2.5. Quantitative Real-Time PCR (qRT-PCR)

At the end of the experiment, cells and tissues of different treatment groups were collected to extract total RNA by TRIzol reagent (Thermo Fisher, no. 15596026). Subsequently, 2 *μ*L of total RNA with a HiFiScript cDNA Synthesis Kit (CWbiotech, no. CW2569) was used to synthesize cDNA. The primers for PAPPA-AS1, GAPDH, U6, TAF15, TLR4, and *β*-actin were designed by using Primer5 software after searching the target gene mRNA sequences on NCBI. The primers for qRT-PCR were listed in Table 1:

We used UltraSYBR Mixture (CWbiotech, no. CW2601) for qRT-PCR, according to the manufacturer's instructions, at 95°C for 10 minutes and the 40 cycles at 95°C for 15 seconds and 60°C for 30 seconds. 2^−ΔΔ*Ct*^ method was used to compare the relative expression of genes.

### 2.6. Western Blotting Assay

Cells were collected and lysed in RIPA lysis buffer (Cell Signaling Technology, Boston, USA). 15% SDS-PAGE was used to segregate the proteins. Subsequently, the isolated proteins were transfer to the PVDF membranes (Cell Signaling Technology, Boston, USA) by semidry transfer. 5–10% BSA solution was added and hatched for 1–2 h. Then, the membranes were incubated with primary antibody against TLR4, MyD88, collagen I, collagen III, TGF-*β*, *α*-SMA, or *β*-actin (1 : 1,000, Abcam, Massachusetts, USA) at 25°C for 2 h. After being washed over, horseradish peroxidase-conjugated secondary antibody (1 : 3,000, Abcam, Massachusetts, USA) was used to incubate with the membranes at 25°C. One to two hours later, blots were incubated with the ECL reagents (Amersham, UK) and exposed under Amersham Imager 600 (GE).

### 2.7. Subcellular Fractionation Assay

We extracted and purified cytoplasmic and nuclear RNAs of the cells through PARIS™ Kit (Invitrogen) according to the manufacturer's instructions. PCR was used to detect RNA content in cytoplasm and nucleus. U6 was used as a nuclear RNA marker and GAPDH as a soluble cytoplasmic marker. The CT value refers to the number of cycles when the fluorescence signal in each reaction tube reaches the set threshold. We used the *Ct* value to measure the content of PAPPA-AS1; the higher the *Ct* value, the lower the content of PAPPA-AS1.

### 2.8. Fluorescence In Situ Hybridization (FISH) Assay

FISH assay was used to detect the localization of PAPPA-AS1 in cells as previously described [27]. PBS was used to wash the cells grown on the slides. 4% paraformaldehyde was used to fix the slides. The images were captured by a confocal microscope.

### 2.9. The Establishment of the HTS Model in Nude Mice

HTS tissues were obtained from clinical HTS patients at least 6 months since the initial injury. Excrescent subcutaneous fat was removed from the human HTS tissues, followed by being cut into 2.0 cm × 1.5 cm slides. The nude mice were made to inhale with 3% isoflurane (tsbiochem, no. T19651) for anesthesia via a nose cone, and a 2.0 cm × 1.5 cm incision on the back of the mouse was clipped out, followed by transplanting the human HTS tissues onto the wounding part and being wrapped. Suture was taken out 15 days postsurgery and the mice with necrotic HTS tissues were excluded from the experiments. All mice were killed by intravenous injection of 150 mg/kg barbiturate. Death was determined by observing the heartbeat and breathing. This experiment has been approved by Xiangya Hospital of Central South University animal experiment ethics committee (no. 2019sydw0009) and Xiangya Hospital of Central South University medical ethics committee (no. 202004121).

### 2.10. Immunofluorescence (IF) Staining

Briefly, after modeling, mice were perfused with cold PBS and then injected with 4% formalin for 24 hours. The isolated HTS tissues were soaked with 4% formalin at 4°C, followed by dehydrating for 7 days using 30% sucrose. Subsequently, the tissues were mounted in OCT and kept in the fridge. 4% paraformaldehyde was used to fix the cells grown on the slides. Primary antibodies rabbit anti-collagen I, anti-collagen III, or anti-*α*-SMA (1 : 200) were used to incubated with the cells or slices prepared by cryosections at a 10 *μ*m thickness. The secondary antibodies conjugated with FITC and DAPI (nuclear marker, color blue) were then incubated for fluorescence staining. The pictures were taken with a fluorescence microscope.

### 2.11. H&E Staining

HTS tissues were isolated from the wounding site of each animal, followed by being washed over by PBS for several hours. After being dehydrated with 70%, 80%, and 90% ethanol solution successively, the tissues were incubated with equal quality of ethanol and xylene. Following being incubated for 15 min, equal quality of xylene was used to mix with the tissues for 15 min. The step was repeated until the tissue looked transparent. Then, the samples were embedded in paraffin, sectioned, and stained with hematoxylin and eosin. The images were caught by an inverted microscope (Olympus, Tokyo, Japan).

### 2.12. Cell Viability Assay

The cell viability was determined by the MTT assay. The cells were collected and incubated in the 96-well plates containing DMEM medium for 24 hours, followed by changing the medium with newly prepared medium containing MTT solution to be incubated for 2 hours at 37°C. Following removing the medium, the MTT solution was dissolved by DMSO at 37°C for 15 min; the absorbance of which was detected at 490 nm according to the protocol described previously [28]. The OD values of each group were recorded to evaluate the cell viability of each group.

### 2.13. Chromatin Immunoprecipitation (ChIP) Assays

The correlation between TAF15 and the promoter region of TLR4 was evaluated by ChIP assay using an EZMagna ChIP kit (Millipore, Billerica, USA). Briefly, the cells were incubated with 1% formaldehyde for 10 min, followed by being terminated with 125 nM glycine. Subsequently, the DNA fragments were extracted from the collected cells at a length between 200 and 1000 bp, followed by being incubated with the specific antibodies at 4°C overnight. Then, DNA enrichment was achieved by adding the Dynabeads Protein G (Thermo Fisher Scientific, Waltham, USA) and incubated for 2 hours. QRT-PCR was used to detect the precipitated DNA and IgG was taken as a negative control in the system.

### 2.14. RNA Pull-Down Assay

The cells were lysed with buffer containing Tris-HCl, EDTA, NaCl, NP-40, protease inhibitor, phosphatase inhibitor, and RNase inhibitor. Followed by centrifugation, the supernatant was collected. A Biotin RNA Labeling Mix kit (Roche, Rotkreuz, Switzerland) was used to biotinylate the extracted RNA, which was then transcribed in vitro. Subsequently, the DNase I (Thermo Fisher Scientific, Waltham, USA) and Sephadex G-50 Quick Spin Columns (Sigma, Missouri, USA) were used to mix with the labeled RNA, which was incubated with the cell supernatant at 4°C for 2 hours. Subsequently, the mixture was incubated with Dynabeads™ MyOne™ Streptavidin T1 (Invitrogen, California, USA) at 4°C for 1 hour. Following being washed over, the SDS-PAGE and Coomassie blue staining were used to detected the proteins with lncRNA PAPPA-AS1. The protein bands were evaluated by the LC-MS/MS mass spectrometry.

### 2.15. Luciferase Reporter Assay

The suspected binding sites of TAF15 in the TLR4 promoter region was cloned into the firefly luciferase in the pGL3 vector (Invitrogen, California, USA). The HTsFb cells were transfected with the plasmids and the TAF15 expressed vector (TAF15 construct) or the negative control (empty vector), which were coincubated with the lipofectamine 3000 (Invitrogen, California, USA) according to the protocol of the manufacturer. Following incubating for 48 hours, the relative luciferase activity was evaluated by the dual-luciferase reporter assay system (Promega, Madison, WI).

### 2.16. RNA Immunoprecipitation (RIP) Assay

The correlation between TLR4 and TAF15 was verified using the EZMagna RIP Kit (Millipore, Massachusetts, USA) according to the manufacturer's instructions. Briefly, the HTsFb cells were lysed with RIP lysis buffer at 4°C half an hour, followed by being incubated with RIP buffer containing magnetic beads, which were conjugated to the antibodies against Ago2 (CST, Boston, USA) or anti-rabbit IgG (negative control, CST, Boston, USA). The precipitated RNAs were analyzed by the RT-qPCR technology and the total RNA was taken as input controls.

### 2.17. Statistical Analysis

All the data in the present study were shown as mean ± SD. GraphPad Prism 8 was used for data analysis. Student's *t*-test was used between the two groups conforming to the normal distribution. Comparisons among multiple groups were conducted by one-way analysis of variance (ANOVA), followed by Tukey's post hoc test. *p* < 0.05 was considered statistically significant.

## 3. Results

### 3.1. lncRNA PAPPA-AS1 Was Screened through Bioinformatics Analysis

**Figure 1:**
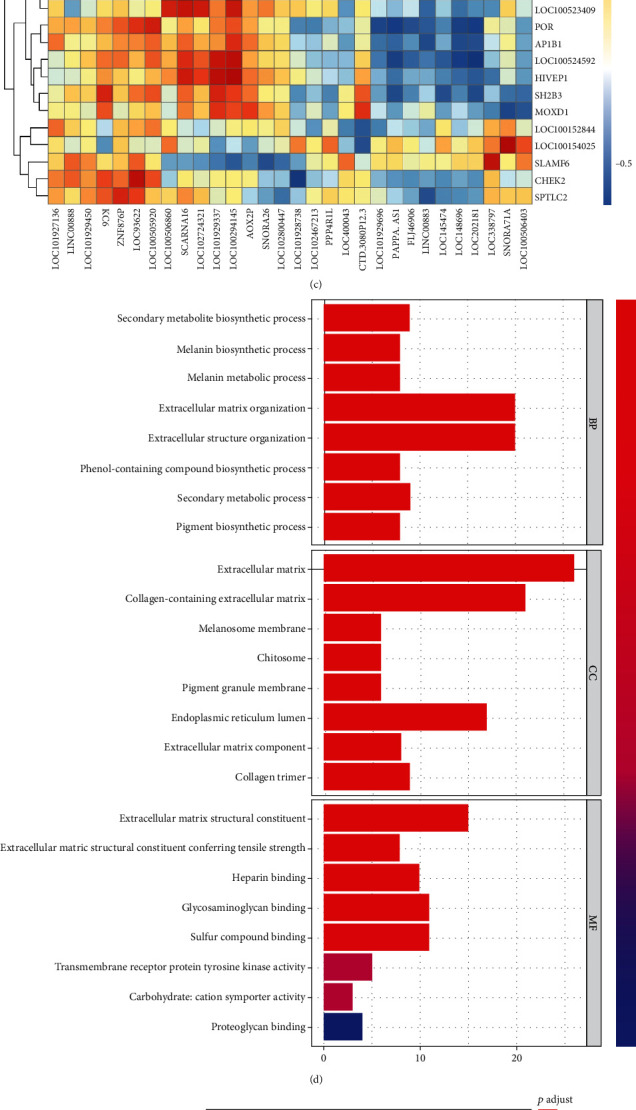
lncRNA PAPPA-AS1 was screened through bioinformatics analysis. (a) Volcano plot of the differentially expressed lncRNAs between HTS and normal skin tissues. (b) Volcano plot of the differentially expressed mRNAs between HTS and normal skin tissues. (c) Correlation clustering heat map of lncRNA and mRNA expression matrix. (d, e) The pathway regulated by mRNA of GO and KEGG enrichment analysis was conducted in HTS tissue.

The GEO dataset was used to screen differentially expressed lncRNA and mRNA in the clinical HTS tissues and the normal skin tissues. As shown in Figure 1(a), lncRNA PAPPA-AS1 was upregulated overtly in the HTS tissues compared with in the normal skin tissues (logFC = 4.46, *p* value < 0.05). Approximately 464 mRNAs were screened out (Figure 1(b)). Among them, 385 and 79 mRNAs were remarkably downregulated and upregulated, respectively, in contrast to the normal skin tissues. In particular, the expression of TLR4, MyD88, and TGF was all significantly upregulated and the multiples of expression difference were 3.26, 2.39, and 2.87 (*p* value < 0.05). The correlation of top 30 upregulated lncRNAs and related mRNA was shown in Figure 1(c). Among them, the expression of lncRNA PAPPA-AS1 was significantly correlated with TLR4, MyD88, and TGF and the expression correlation was greater than 0.5. We selected the evidently upregulated mRNAs for functional enrichment analysis. The results of Go enrichment analysis showed that the mRNAs played important roles in various biological processes, including the secondary metabolite biosynthetic process, melanin biosynthetic process, melanin metabolic process, extracellular matrix, collagen-containing extracellular matrix, melanosome membrane, extracellular matrix structural constituent, extracellular matrix structural constituent conferring tensile strength, and heparin binding (Figure 1(d)). The results of KEGG analysis suggested the mRNAs were mainly associated with the PI3K-Akt signaling pathway, protein digestion and absorption, and cell adhesion molecules (Figure 1(e)). Therefore, we speculated that the development of HTS was related to the abnormal activation of lncRNA PAPPA-AS1 and the TLR4/MyD88/TGF signaling pathway.

### 3.2. lncRNA PAPPA-AS1 Was Significantly Upregulated in Clinical HTS Tissues and HTsFb Cells

**Figure 2:**
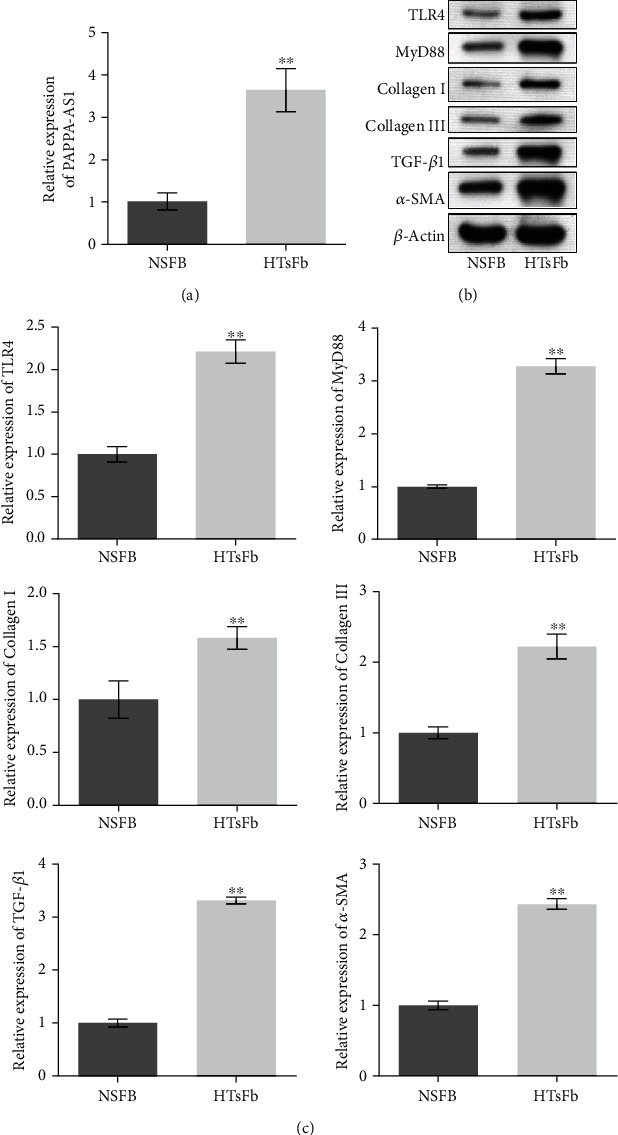
lncRNA PAPPA-AS1 was significantly upregulated in clinical HTS tissues and HTsFb cells. (a) qRT-PCR was used to detect the expression of PAPPA-AS1 in the NsFb and HTsFb cells. (b, c) The expression levels of TLR4, MyD88, collagen I, collagen III, TGF-*β*1, and *α*-SMA were evaluated by Western blot. NSFB group: normally cultured NSFB cells; HTsFb group: normally cultured HTsFb cells (^∗∗^*p* < 0.01 vs. NsFb).

To verify the relative expression level of these lncRNAs in HTS and pick out the most significant one, the expression of these lncRNAs in the NsFb and HTsFb cells was evaluated. As shown in Figure 2(a), approximately 3-fold expressional change on lncRNA PAPPA-AS1 was observed in HTsFb cells, compared to NsFb cells (^∗∗^*p* < 0.01 vs. NsFb), which was the lncRNA with the most significance. To evaluate the state of the TLR4 downstream signal pathway, the expression level of related proteins was determined in both the NsFb and HTsFb cells. As shown in Figures 2(b) and 2(c), we found that TLR4, MyD88, collagen I, collagen III, TGF-*β*1, and *α*-SMA were significantly upregulated in the HTsFb cells, compared to NsFb cells (^∗∗^*p* < 0.01 vs. NsFb).

### 3.3. Knocking Down PAPPA-AS1 Suppressed the Proliferation of HTsFb Cells by Inhibiting the TLR4/MyD88 and TGF-*β*1 Signal Pathways In Vitro

**Figure 3:**
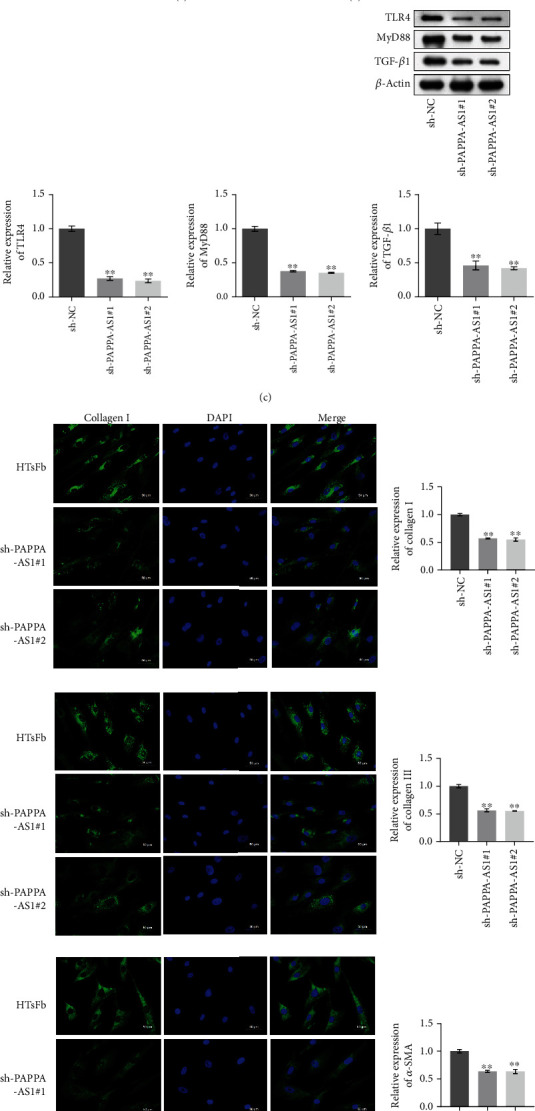
Knocking down PAPPA-AS1 suppressed the proliferation of HTsFb cells by inhibiting the TLR4/MyD88 and TGF-*β*1 signal pathways. (a) QRT-PCR was used to detect the expression of PAPPA-AS1. (b) MTT assay was used to check the cell proliferation ability. (c) The expression levels of TLR4, MyD88, and TGF-*β*1 were evaluated by Western blot. (d) The expression levels of collagen I, collagen III, and *α*-SMA were evaluated by IF staining. sh-NC group: HTsFb cells were transfected with sh-NC plasmid; sh-PAPPA-AS1#1 group: HTsFb cells were transfected with sh-PAPPA-AS1#1 plasmid; sh-PAPPA-AS1#2 group: HTsFb cells were transfected with sh-PAPPA-AS1#2 plasmid (^∗^*p* < 0.05 vs. sh-NC, ^∗∗^*p* < 0.01 vs. sh-NC).

To evaluate the function of PAPPA-AS1 in HTsFb cells, the PAPPA-AS1 knockdown HTsFb cells were established by short hairpin RNA (shRNA) technology. As shown in Figure 3(a), the expression of PAPPA-AS1 was significantly suppressed in the sh-PAPPA-AS1#1 or sh-PAPPA-AS1#2 group, compared with the sh-NC group (^∗∗^*p* < 0.01 vs. sh-NC). We further detected the proliferation ability of transfected cells. As shown in Figure 3(b), the OD value in the sh-PAPPA-AS1#1 or sh-PAPPA-AS1#2 group was significantly lower, compared with the sh-NC group (^∗∗^*p* < 0.01 vs. sh-NC), indicating an inhibitory effect on HTsFb cell proliferation ability by knocking down PAPPA-AS1. In addition, by transfecting the HTsFb cells with sh-PAPPA-AS1#1 or sh-PAPPA-AS1#2, the expression levels of TLR4, MyD88, and TGF-*β*1 (Figure 3(c)) in the HTsFb cells were significantly suppressed (^∗∗^*p* < 0.01 vs. sh-NC).  Figure 3(d) showed the fluorescence intensities of fibrosis markers, collagen I, collagen III, and *α*-SMA, in the treated HTsFb cells. We found that the fluorescence intensities of collagen I, collagen III, and *α*-SMA decreased greatly in the sh-PAPPA-AS1#1 or sh-PAPPA-AS1#2 group, compared with the sh-NC group.

### 3.4. Knocking Down PAPPA-AS1 Alleviated the Fibrosis State of HTS Mice In Vivo

**Figure 4:**
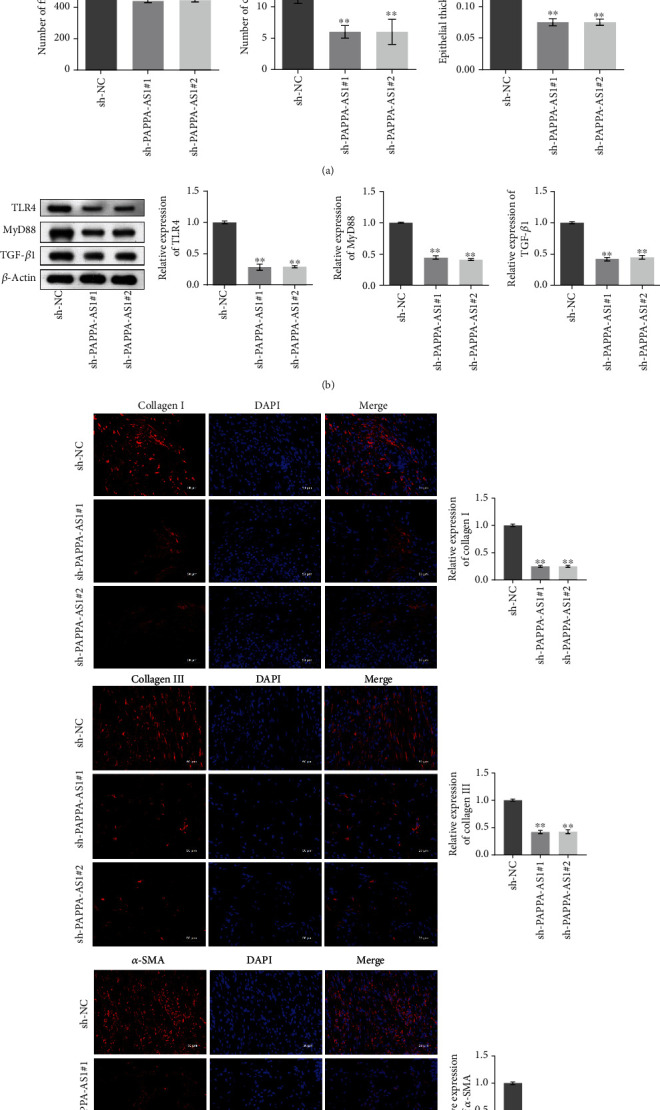
Knocking down PAPPA-AS1 alleviated the fibrosis state of HTS mice. (a) HE staining was used to evaluate the pathological state of HTS tissues, and the number of fibroblasts, number of capillaries, and epithelial thickness were counted (^∗∗^*p* < 0.01 vs. sh-NC). (b) The expression levels of TLR4, MyD88, and TGF-*β*1 in the HTS tissues were evaluated by Western blot (^∗∗^*p* < 0.01 vs. sh-NC). (c) The expression levels of collagen I, collagen III, and *α*-SMA in the HTS tissues were evaluated by IF staining. sh-NC group: mice were injected with sh-NC lentivirus; sh-PAPPA-AS1#1 group: mice were injected with sh-PAPPA-AS1#1 lentivirus; sh-PAPPA-AS1#2 group: mice were injected with sh-PAPPA-AS1#2 lentivirus.

To evaluate the effect of lncRNA PAPPA-AS1, we inserted the shRNAs into the lentiviral particles, which were further injected into the HTS tissues. As shown in Figure 4(a), compared with sh-NC, the number of fibroblasts, the number of capillaries, and epithelial thickness were decreased in the sh-PAPPA-AS1#1 and sh-PAPPA-AS1#2 groups. In addition, the expression levels of TLR4, MyD88, and TGF-*β*1 (Figure 4(b)) in the HTS tissues were significantly suppressed in the sh-PAPPA-AS1#1 or sh-PAPPA-AS1#2 group, compared with the sh-NC group (^∗∗^*p* < 0.01 vs. sh-NC).  Figure 4(c) showed the fluorescence intensities of collagen I, collagen III, and *α*-SMA in the HTS tissues of each group. The fluorescence intensities of collagen I, collagen III, and *α*-SMA were suppressed greatly in the sh-PAPPA-AS1#1 or sh-PAPPA-AS1#2 group, compared with the sh-NC group.

### 3.5. lncRNA PAPPA-AS1 Interacted with TAF15 and Upregulated Its Expression in the HTsFb Cells In Vitro

**Figure 5:**
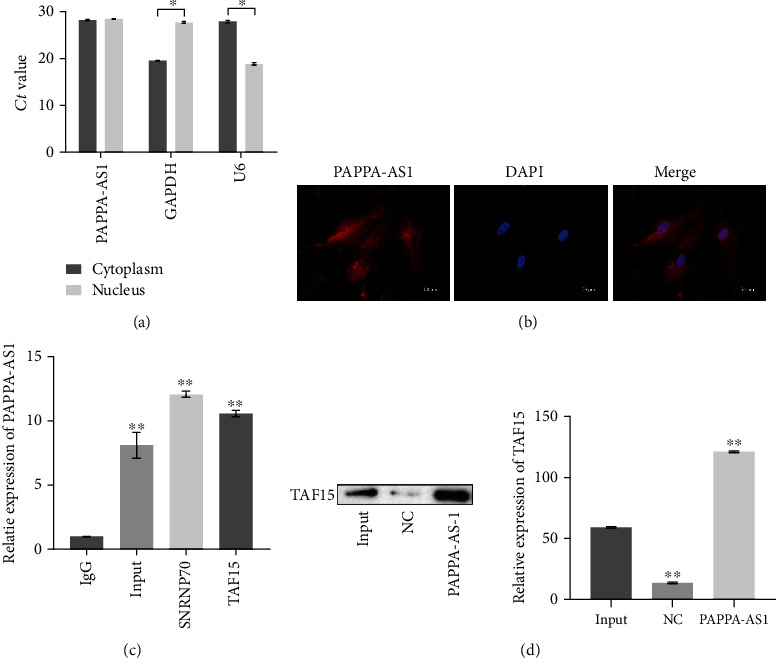
lncRNA PAPPA-AS1 interacted with TAF15 and upregulated its expression in the HTsFb cells. (a) Subcellular fractionation assay was used to determine the expression of PAPPA-AS1 in the cytoplasm and nucleus (^∗^*p* < 0.05 vs. cytoplasm). (b) The expression of PAPPA-AS1 in the cytoplasm and nucleus was verified by RNA FISH assay. (c, d) The interaction between PAPPA-AS1 and TAF15 was detected by RIP assay and RNA pull-down assay (^∗∗^*p* < 0.01 vs. IgG).

To explore the mechanism underlying the regulatory effect of lncRNA PAPPA-AS1 on the TLR4/MyD88 and TGF-*β*1 signal pathways, we explored the interaction of lncRNA PAPPA-AS1 with an RNA-binding protein (RBP), TAF15. As shown in Figures 5(a) and 5(b), we found that lncRNA PAPPA-AS1 was located both in the nucleus and cytoplasm of the HTsFb cells. The results of RIP assay were shown in Figure 5(c). Taking SNRNP70 as a positive control, compared to IgG, lncRNA PAPPA-AS1 was significantly highly expressed in the TAF15 group. To further confirm the interaction between lncRNA PAPPA-AS1 and TAF15, we performed the RNA pull-down assay. As shown in Figure 5(d), the results showed that lncRNA PAPPA-AS1 interacted with TAF15, indicating an obvious interaction between lncRNA PAPPA-AS1 and TAF15 (^∗∗^*p* < 0.01 vs. IgG).

### 3.6. lncRNA PAPPA-AS1 Induced the Transcription of TLR4 by Recruiting TAF15 to the TLR4 Promoter In Vivo and Vitro

**Figure 6:**
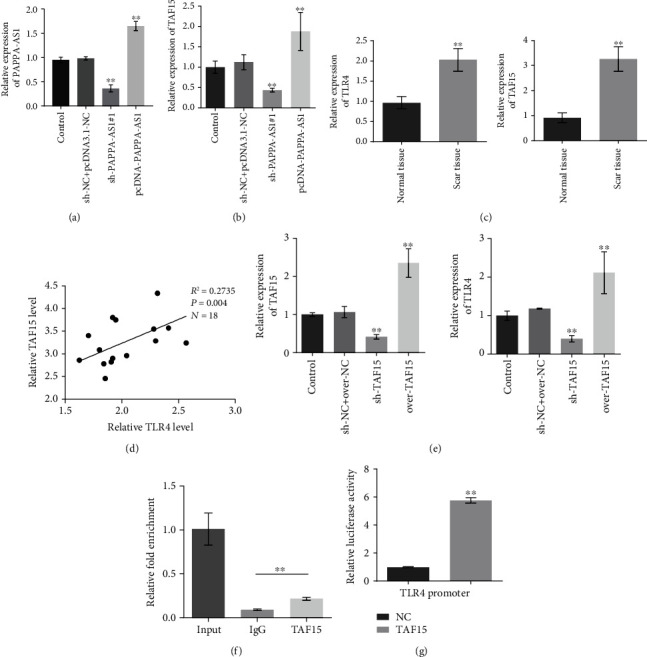
lncRNA PAPPA-AS1 induced the transcription of TLR4 by recruiting TAF15 to the TLR4 promoter. (a) The relative expression of PAPPA-AS1 was detected by qRT-PCR. (b) qRT-PCR was used to detect the expression of TAF15 (^∗∗^*p* < 0.01 vs. sh − NC + pcDNA3.1 − NC). (c) The expression levels of TAF15 and TLR4 in the tissues were evaluated by RT-qPCR (^∗∗^*p* < 0.01 vs. normal tissues). (d) The correlation between the expression of TAF15 and TLR4 in the HTS tissues was evaluated. (e) RT-qPCR was used to detect the expression of TAF15 and TLR4 in the treated HTsFb cells (^∗∗^*p* < 0.01 vs. sh − NC + over − NC). (f) ChIP assay evaluated the interaction between TAF15 and the promoter of TLR4 (^∗∗^*p* < 0.01 vs. IgG). (g) The interaction between TAF15 and the promoter of TLR4 was verified by luciferase assay. Control group: normally cultured HTsFb cells; sh − NC + pcDNA3.1 − NC group: HTsFb cells were transfected with sh-NC plasmid and pcDNA3.1-NC plasmid simultaneously; sh-PAPPA-AS1#1 group: HTsFb cells were transfected with sh-PAPPA-AS1#1 plasmid; pcDNA3.1-PAPPA-AS1 group: HTsFb cells were transfected with pcDNA3.1-PAPPA-AS1 plasmid; normal tissue group: human normal skin tissue; scar tissue: human scar tissue; sh − NC + over − NC group: HTsFb cells were transfected with sh-NC plasmid and over-NC plasmid simultaneously; sh-TAF15 group: HTsFb cells were transfected with sh-TAF15 plasmid; over-TAF15 group: HTsFb cells were transfected with over-TAF15 plasmid. (^∗∗^*p* < 0.01 vs. NC).

As the knockdown efficacy was nearly equal between sh-PAPPA-AS1#1 and sh-PAPPA-AS1#2, one of the shRNAs was chosen for the subsequent experiments (sh-PAPPA-AS1#1). In addition, PAPPA-AS1-overexpressed HTsFb cells were established by transfecting the cells with the pcDNA3.1-PAPPA-AS1 vector. It showed that the transfections of the overexpression of PAPPA-AS1 were successful (Figure 6(a)). As shown in Figure 6(b), compared to sh − NC + pcDNA − 3.1 − NC, TAF15 was significantly downregulated in the sh-PAPPA-AS1#1 group and upregulated in the over-PAPPA-AS1 group, indicating a positive correlation between the expression of PAPPA-AS1 and TAF15. As shown in the aforementioned results, the TLR4 signal pathway was regulated by lncRNA PAPPA-AS1, which might be related to the development of HTS. We further investigated the expression levels of TLR4 and TAF15 in the normal skin tissues and the scar tissues. Both TLR4 and TAF15 were found to be significantly upregulated in the scar tissues (Figure 6(c), ^∗∗^*p* < 0.01 vs. normal tissues). In addition, a positive correlation between the expression levels of TLR4 and TAF15 in the scar tissues was observed (Figure 6(d)). To explore the relationship between TLR4 and TAF15, the TAF15 knockdown and TAF15-overexpressed HTsFb cells were established. As shown in Figure 6(e), the expression level of TAF15 was significantly suppressed in the sh-TAF15 group and elevated in the over-TAF15 group. In addition, TLR4 was downregulated in the sh-TAF15 group and upregulated in the over-TAF15 group (^∗∗^*p* < 0.01 vs. control). ChIP assay (Figure 6(f)) revealed that TAF15 interacted with the promoter of TLR4 (^∗∗^*p* < 0.01 vs. IgG). As shown in Figure 6(g), significant luciferase activity was observed in the TAF15-transfected cells, further verifying the interaction between TAF15 and TLR4 (^∗∗^*p* < 0.01 vs. NC).

### 3.7. PAPPA-AS1 Promoted the Proliferation of HTsFb Cells by Regulating the TAF15/TLR4 Axis In Vitro

**Figure 7:**
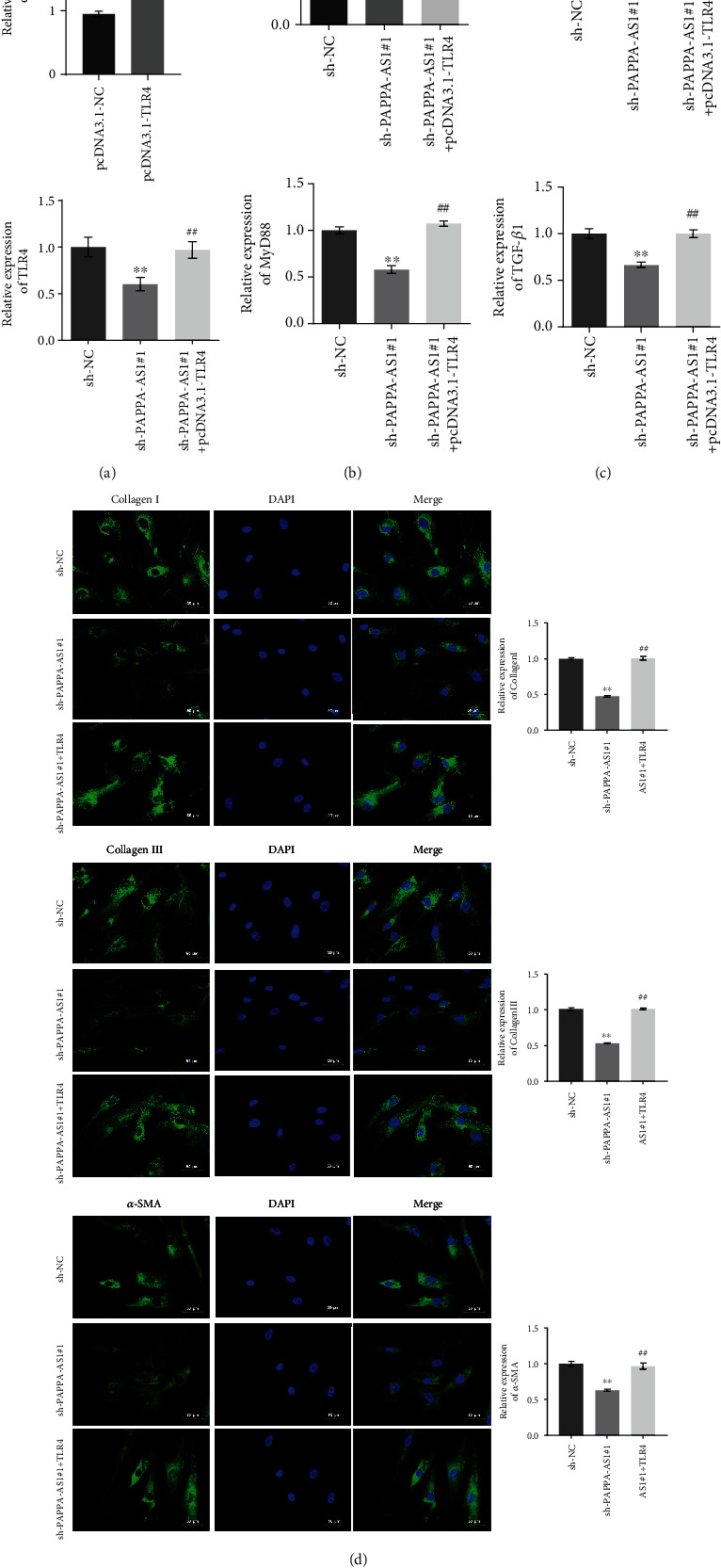
PAPPA-AS1 promoted the proliferation of HTsFb cells by regulating the TAF15/TLR4 axis. (a) The relative expression of TLR4 was detected by qRT-PCR. (b) MTT assay was used to detect the proliferation of the treated HTsFb cells (^∗^*p* < 0.05, vs. sh-NC; ^##^*p* < 0.01 vs. sh-PAPPA-AS1#1). (c) The expression levels of TLR4, MyD88, and TGF-*β*1 were evaluated by Western blot. (d) The expression levels of collagen I, collagen III, and *α*-SMA were evaluated by IF staining. pcDNA3.1-NC group: HTsFb cells were transfected with pcDNA3.1-NC plasmid; pcDNA3.1-TLR4 group: HTsFb cells were transfected with pcDNA3.1-TLR4 plasmid; sh-NC group: HTsFb cells were transfected with sh-NC plasmid; sh-PAPPA-AS1#1 group: HTsFb cells were transfected with sh-PAPPA-AS1#1 plasmid; sh − PAPPA − AS1#1 + pcDNA3.1 − TLR4 group: HTsFb cells were transfected with sh-PAPPA-AS1#1 and pcDNA3.1-TLR4 plasmid simultaneously (^∗∗^*p* < 0.01 vs. sh-NC, ^##^*p* < 0.01 vs. sh-PAPPA-AS1#1).

After confirming the interaction between lncRNA PAPPA-AS1 and TAF15, as well as TAF15 and TLR4, we further verify whether the regulatory effect of PAPPA-AS1 on the proliferation was related to the impact of PAPPA-AS1 on TLR4 expression. In order to verify the success of TLR4 overexpression, we carried out qRT-PCR detection.  Figure 7(a) showed that the expression of TLR4 in HTsFb cells increased after transfection of pcDNA-TLR4. The PAPPA-AS1 knockdown HTsFb cells, and the PAPPA-AS1 knockdown and TLR4-overexpressed HTsFb cells were established. As shown in Figure 7(b), the suppressed cell viability of HTsFb cells in the sh-PAPPA-AS1#1 group was significantly elevated in the sh − PAPPA − AS1#1 + TLR4 group (^∗∗^*p* < 0.01 vs. sh-NC, ^##^*p* < 0.01 vs. sh-PAPPA-AS1#1). In addition, the downregulated TLR4, MyD88, and TGF-*β*1 induced by the knockdown of PAPPA-AS1 were greatly reversed in the sh − PAPPA − AS1#1 + pcDNA − TLR4 group (Figure 7(c), ^∗∗^*p* < 0.01 vs. sh-NC, ^##^*p* < 0.01 vs. sh-PAPPA-AS1#1). As shown in Figure 7(d), compared with sh-NC group, fluorescence intensities of collagen I, collagen III, and *α*-SMA were suppressed greatly in the sh-PAPPA-AS1#1 group, which was significantly elevated in the sh − PAPPA − AS1#1 + pcDNA − TLR4 group.

### 3.8. PAPPA-AS1 Impacted the Fibrosis State of HTS Mice by Regulating the TAF15/TLR4 Axis In Vivo

**Figure 8:**
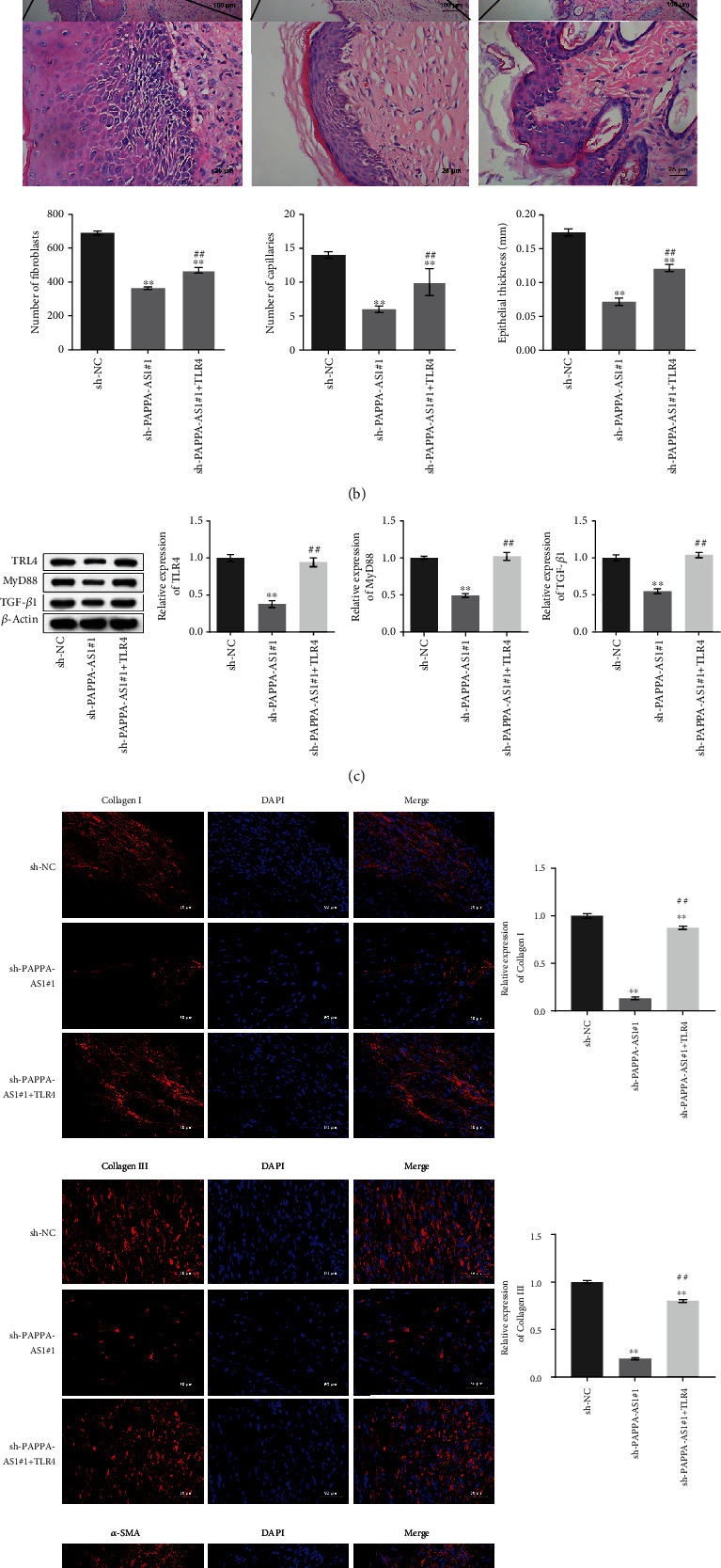
PAPPA-AS1 impacted the fibrosis state of HTS mice by regulating the TAF15/TLR4 axis. (a) The relative expression of TLR4 was detected by qRT-PCR. (b) HE staining was used to evaluate the pathological state of HTS tissues. (c). The expression levels of TLR4, MyD88, and TGF-*β*1 in the HTS tissues were evaluated by Western blot (^∗∗^*p* < 0.01 vs. sh-NC, ^##^*p* < 0.01 vs. sh-PAPPA-AS1#1). (d) The expression levels of collagen I, collagen III, and *α*-SMA in the HTS tissues were evaluated by IF staining. pcDNA3.1-NC group: mice were injected with pcDNA3.1-NC lentivirus; pcDNA3.1-TLR4 group: mice were injected with pcDNA3.1-TLR4 lentivirus; sh-NC group: mice were injected with sh-NC lentivirus; sh-PAPPA-AS1#1 group: mice were injected with sh-PAPPA-AS1#1 lentivirus; sh − PAPPA − AS1#1 + pcDNA3.1 − TLR4 group: mice were injected with sh-PAPPA-AS1#1 and pcDNA3.1-TLR4 lentivirus simultaneously.

To verify the underlying mechanism on the function of PAPPA-AS1 in the development of HTS, sh-PAPPA-AS1#1 and sh-PAPPA-AS1#1 combined with the pcDNA3.1-TLR4 vector were inserted into the lentiviral particles, which were further injected into the HTS tissues of the animals. The results in Figure 7(a) show that the expression of TLR4 in the pcDNA-TLR4 group was higher than that in the pcDNA-NC group. It was suggested that pcDNA-TLR4 played an important role. As shown in Figure 8(b), compared with the sh-NC group, the number of fibroblasts and the number of capillaries were reduced in the sh-PAPPA-AS1#1 group, which were reversed in the sh − PAPPA − AS1#1 + pcDNA − TLR4 group, and so as the epithelial thickness. The inhibited expression levels of TLR4, MyD88, and TGF-*β*1 in the HTS tissues (Figure 8(c)) induced by knockdown of PAPPA were significantly elevated in the sh − PAPPA − AS1#1 + pcDNA − TLR4 group (^∗∗^*p* < 0.01 vs. sh-NC and ^##^*p* < 0.01 vs. sh-PAPPA-AS1#1). In addition, the decreased fluorescence intensities of collagen I, collagen III, and *α*-SMA in the HTS tissues induced by knockdown of PAPPA were significantly elevated in the sh − PAPPA − AS1#1 + pcDNA − TLR4 group (Figure 8(d)).

## 4. Discussion

lncRNA has been reported to be involved in multiple types of diseases by regulating the expression of key proteins, including malignant tumors [29, 30], fibrosis [31, 32], and autoimmune diseases [33, 34]. Jiang and Guo found that THBS1 induced the growth of hypertrophic scar fibroblasts [35]. HTS is a complicated pathological process with multiple cellular signal pathways and abnormally expressed proteins involved; the development and process of which are recently reported to be related to the differentially expressed lncRNA [36]. Ma and Liu claimed that lncRNA FAM225B promoted the progression of HTS [37]. However, the underlying mechanism is still unknown. In the present study, the differentially expressed lncRNAs between normal skin tissues and HTS tissues were screened using a lncRNA array. lncRNA PAPPA-AS1 was picked out due to its high expression level in the HTsFb cells compared to NsFb. Fibroblasts are reported to be the main participants in the process and development of HTS by secreting collagens; the excessive proliferation of which is regarded as the main inducer for the formation of HTS [38, 39]. Therefore, in the present study, HTsFb cells were taken as the study objects. By knocking down the expression level of lncRNA PAPPA-AS1 in the HTsFb cells, the proliferation ability of HTsFb cells and the expression level of fibrosis markers were significantly suppressed, indicating a potential correlation between lncRNA PAPPA-AS1 and the fibrosis process of HTsFb cells. In addition, the in vivo experiments confirmed that the pathological state of HTS tissues was alleviated and the expression level of fibrosis markers was inhibited by injecting the lentiviral particles containing the shRNA against lncRNA PAPPA-AS1. Taken these preliminary data together, we suspected that the development of HTS was possibly related to the upregulation of lncRNA PAPPA-AS1. But we still do not know what kinds of molecules induced lncRNA PAPPA-AS1 constitutively in HTsFb. In the future, we will use bioinformatics methods to explore and predict the molecules acting on PAPPA-AS1 and provide more possibilities for better HTS treatment.

The TLR4/MyD88 signal pathway is a classic inflammatory signal pathway, which is reported to exert an important role in the development and process of fibrosis [40]. By regulating the NF-*κ*B signal pathway, the TLR4/MyD88 signal triggers the excessive production of inflammatory factors to induce and aggravate the pathological state of fibrosis [41, 42]. In addition, the TGF-*β*1 signal pathway can be activated by the stimulation on the TLR4/MyD88 axis [19], which is regarded as an important growth factor in fibrogenesis. By inducing the formation of ECM and preventing ECM from being degraded, the production of fibrogenic myofibroblasts will be inhibited by TGF-*β*1 [43]. In addition, the regulatory effect of TGF-*β*1 on fibrogenic activities was related to the activation of Smad signaling pathways through impacting the type I receptor-mediated phosphorylation of Smad2 and Smad3 [44]. Suppressing TGF-*β*1 has been proved to alleviate fibrosis in multiple animal models [45, 46]. In the present study, we found that both the TLR4/MyD88 signal pathway and the TGF-*β*1 signal pathway were significantly activated in the HTsFb cells, which were reversed by knocking down the expression level of lncRNA PAPPA-AS1, indicating that lncRNA PAPPA-AS1 might induce the process of HTS by suppressing the TLR4/MyD88 signal pathway and TGF-*β*1 signal pathway. However, there may be other molecules in the TLR4/MyD88 pathway and we still cannot conclude that it is entirely the role of TLR4. TLR4 may play more complex functions in scars, which requires a more comprehensive research to explore. Therefore, we will carry out thorough analysis and complete experiments to explore the role of various molecules in the TLR4/MyD88 pathway on HTS in our future work. We will also combine the current work to compare the difference between TLR4 alone and its collaboration function with PAPPA-AS1.

To further investigate the molecular mechanism, we focused on the RNA-binding protein, TATA-box binding protein-associated factor 15 (TAF15), which belongs to the FET family [47]. The FET family is widely expressed in different tissues, and the structure of the family members is highly similar to each other, which is reported to be involved in such biological processes as transcription, splicing, miRNA formation, RNA transport, and maintenance of genomic integrity [48]. Recently, TAF15 is widely reported to interact with lncRNA to regulate the transcription and translation of target genes. Pan et al. [49] claims that in the process of colon cancer, lncRNA TRPM2-AS interacts with TAF15 to stabilize the mRNA of transient receptor potential melastatin 2 (TRPM2), which induces the development of colon cancer. The expression level of LINC010148 can be elevated by USF1, and LINC010148 interacts with TAF15 to bind to the promoter of YAP1, which further triggers the development of squamous carcinoma [50]. In the present study, the interaction between lncRNA PAPPA-AS1 and TAF15 was confirmed by RNA pull-down and RIP assay. In addition, the binding between TAF15 and the promoter of TLR4 was confirmed by ChIP and luciferase assay. We further verified that lncRNA PAPPA-AS1 regulated the TLR4/MyD88 signal pathway and TGF-*β*1 signal pathway in the HTsFb cells by interacting with TAF15 to upregulate the expression level of TLR4, which was further proved in the HTS nude mouse model.

However, there was still limitation in the present study. For example, we verified the function of the lncRNA PAPPA-AS1/TAF15/TLR4 axis by transfecting both sh-PAPPA-AS1#1 and pcDNA3.1-TLR4 into HTsFb cells or HTS tissues, which abolished the effects of sh-PAPPA-AS1#1. No direct evidence was provided to claim the involvement of TAF15. In our future work, upregulating TAF15 in the sh-PAPPA-AS1#1-transfected HTsFb cells or HTS tissues will be performed to directly verify the involvement of TAF15. In addition, in the present study, the xenograft HTS tissues from the clinic were transplanted onto the nude mice to establish the animal model, which was independent and only provided the prospective data. In our further work, other HTS animal models, such as a HTS rabbit model and transgenic animals, will be used to evaluate the biofunction of lncRNA PAPPA-AS1 and the potential underlying mechanism in the development and process of HTS.

## 5. Conclusion

Through our experiments and analysis, we demonstrated that lncRNA PAPPA-AS1 might induce the development of HTS by upregulating TLR4 through interacting with TAF15.

## Figures and Tables

**Table 1:** Primers for genes involved in qRT-PCR.

Gene	Primer
PAPPA-AS1 RT	Forward 5′-AGCCTCTTTTGCCTAATATCCTT-3′
	Reverse 5′-GCCACAGAAGAACCTTACCAG-3′
GAPDH RT	Forward 5′-ACAGCCTCAAGATCATCAGC-3′
	Reverse 5′-GGTCATGAGTCCTTCCACGAT-3′
U6 RT	Forward 5′-CTCGCTTCGGCAGCACA-3′
	Reverse 5′-AACGCTTCACGAATTTGCGT-3′
TAF15 RT	Forward 5′-ACAAGGACACAGGAAAGCCAAA-3′
	Reverse 5′-AATTCAGGTCTTCTAGTGGCAA-3′
TLR4 RT	Forward 5′-AGACACTTTATTCAGAGCCGTTG-3′
	Reverse 5′-AAGGCGATACAATTCCACC-3′
*β*-Actin RT	Forward 5′-ACCCTGAAGTACCCCATCGAG-3′
	Reverse 5′-AGCACAGCCTGGATAGCAAC-3′

## Data Availability

The datasets used or analyzed during the current study are available from the corresponding author on reasonable request.
